# TGFB1-Mediated Gliosis in Multiple Sclerosis Spinal Cords Is Favored by the Regionalized Expression of HOXA5 and the Age-Dependent Decline in Androgen Receptor Ligands

**DOI:** 10.3390/ijms20235934

**Published:** 2019-11-26

**Authors:** Serge Nataf, Marine Guillen, Laurent Pays

**Affiliations:** 1 Bank of Tissues and Cells, Lyon University Hospital (Hospices Civils de Lyon), 69003 Lyon, France; laurent.pays@univ-lyon1.fr; 2 CarMeN Laboratory, INSERM 1060, INRA 1397, INSA Lyon, 69600 Oullins, France; 3 University Claude Bernard Lyon-1, F-69000 Lyon, France; marine.guillen@univ-lyon1.fr

**Keywords:** multiple sclerosis, spinal cord, transforming growth factor beta 1, astrocytes, androgen receptor, homeobox A5

## Abstract

In multiple sclerosis (MS) patients with a progressive form of the disease, spinal cord (SC) functions slowly deteriorate beyond age 40. We previously showed that in the SC of these patients, large areas of incomplete demyelination extend distance away from plaque borders and are characterized by a unique progliotic TGFB1 (Transforming Growth Factor Beta 1) genomic signature. Here, we attempted to determine whether region- and age-specific physiological parameters could promote the progression of SC periplaques in MS patients beyond age 40. An analysis of transcriptomics databases showed that, under physiological conditions, a set of 10 homeobox (HOX) genes are highly significantly overexpressed in the human SC as compared to distinct brain regions. Among these HOX genes, a survey of the human proteome showed that only HOXA5 encodes a protein which interacts with a member of the TGF-beta signaling pathway, namely SMAD1 (SMAD family member 1). Moreover, HOXA5 was previously found to promote the TGF-beta pathway. Interestingly, SMAD1 is also a protein partner of the androgen receptor (AR) and an unsupervised analysis of gene ontology terms indicates that the AR pathway antagonizes the TGF-beta/SMAD pathway. Retrieval of promoter analysis data further confirmed that AR negatively regulates the transcription of several members of the TGF-beta/SMAD pathway. On this basis, we propose that in progressive MS patients, the physiological SC overexpression of HOXA5 combined with the age-dependent decline in AR ligands may favor the slow progression of TGFB1-mediated gliosis. Potential therapeutic implications are discussed.

## 1. Introduction

Magnetic resonance imaging (MRI) studies have demonstrated that spinal cord (SC) tissue alterations correlate with clinical disability in progressive forms of multiple sclerosis (MS), be it secondary progressive MS (SPMS) or primary progressive MS (PPMS) [1,2,3]. Importantly, in PPMS or SPMS, scores of clinical disability tend to inexorably progress starting from the fourth decade of life [4,5], and this occurs despite an overall decrease in the number of active inflammatory lesions in the brain [6,7]. In this context, we recently reported on the in-depth analysis of SC molecular neuropathology in SPMS or PPMS patients. We found that areas of incomplete demyelination extend distance away from plaque borders [8] and are characterized by a TGF-beta 1 (transforming growth factor beta 1) progliotic signature [9]. Based on the identification of astrocyte vs. oligodendrocyte gene co-expression networks, we further proposed that TGF-beta 1, while preventing acute inflammation, could (i) promote gliosis, (ii) prevent the antigliotic effects mediated by androgen receptor ligands, and (iii) alter the translation of myelin genes [9]. However, the question as to whether such a process could be, to some extent, SC-specific, was not assessed.

Interestingly, to our best knowledge, only one article has reported on an extensive and systematic neuropathological analysis of plaque activity on both the brains and SCs derived from a large cohort of MS patients [10]. While the main conclusion of this work related with the role of smoldering plaques (i.e., slowly expanding lesions) in MS progression, an important finding was unraveled but somehow neglected and not discussed. Indeed, when assessing the impact of localization on the percentage of active vs. inactive plaques, the authors found that irrespective of age and disease duration, significant differences were observed when comparing the SC to the brain: “Lesions in the SC were more likely to be inactive (*p* < 0.001, *p* = 0.002) compared to supratentorial and infratentorial lesions. In addition “Lesions in the SC were less likely to be smoldering (*p* = 0.02) compared to supratentorial lesions” [10]. Finally, “no/few smoldering plaques were found in the SC or optic nerve” while “smoldering and inactive plaques were both equally distributed between the supratentorial and the infratentorial white matter” [10]. Importantly, the authors also reported that active plaques did not display any region-specific distribution even when specifically assessing early active or late active plaques [10]. It is worth noting that, although based on the analysis of fewer samples, a previous work similarly concluded a dissociation between brain and SC neuropathological features in SPMS or PPMS patients. Such a dissociation was reported with regard to both the percentage of inactive plaques (89% of inactive plaques in the SC as compared to 54% in the brain) and the percentage of slowly expanding plaques (5% of slowly expanding plaques in the SC as compared to 18% in the brain) [11]. It appears thus that downstream of the triggering autoimmune mechanisms leading to the formation of active plaques, an SC-specific process might be responsible for dampening of plaque-associated inflammation. If so, myelin repair, a process known to be coupled with plaque-associated inflammatory events, would also differ between the brain and SC. In this functional scheme, the identification of a TGFB1 genomic signature in MS spinal cords makes sense since TGFB1 was demonstrated to dampen acute central nervous system (CNS) inflammatory lesions [12,13] to exert potent progliotic effects (notably via the astrocytic synthesis of extracellular matrix molecules) [14,15,16] and to both inhibit the terminal differentiation of oligodendrocyte progenitors and prevent microglia-mediated remyelination [17].

In the present paper, we mined transcriptomics and proteomics databases to identify physiological parameters that would be responsible for a region-specific and age-dependent susceptibility of human SC to TGFB1-mediated gliosis. Our results may explain the particular outcome of SC active plaques and in progressive MS patients, the age-dependent deterioration of SC functions. 

## 2. Results

### 2.1. The Human Spinal Cord Genomic Signature Retrieved from the ARCHS4 Database Is Specifically Enriched in Homeobox Genes

**Figure 1:**
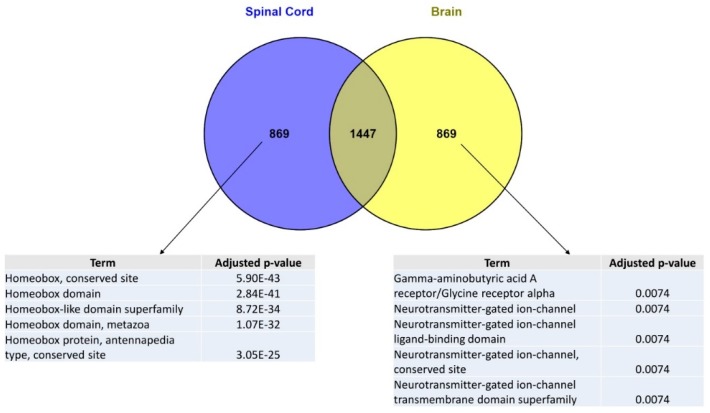
Enrichment analyses of spinal cord vs. brain genomic signatures. The genomic signatures of human brain and spinal cord were retrieved from the “ARCHS4 Tissue” [19] library gathering and combining 84,863 publicly available human RNA-seq data for which gene expression values were z-score normalized across samples. Brain and spinal cord genomic signatures were compared and crossed in order to identify spinal cord- vs. brain-specific signatures. The retrieved lists of genes were then submitted to an enrichment analysis using the TargetMine [20] webtool “InterPro domain” which exploits the InterPro protein families database [21]. The TargetMine bioinformatics platform provides *p*-values computed from the Fisher’s exact test and adjusted for multiple test corrections using the Benjamini and Hochberg procedure. For each list, only the 5 most statistically significant enrichments are shown.

In order to identify genes whose expression is SC-specific as compared to other CNS regions, we first explored the ARCHS4 library of tissue-specific genomic signatures which may be accessed via the Enrichr platform [18]. The ARCHS4 library, obtained by the combined analysis of 84,863 publicly available human RNA-seq data, gathers genomic signatures for 108 human tissues or cell types, irrespective of the presence or absence of a pathological state [19]. From the ARCHS4 library, we retrieved brain and spinal cord genomic signatures and extracted two sets of genes specific to each of these signatures. These two lists of genes were then submitted to enrichment analyses via the TargetMine platform [20]. Interestingly, the most significant enrichment was obtained using the InterPro domain enrichment tool [21]. Indeed, we found that the 869 genes which are specific to the SC signature (as compared to the brain signature) were highly significantly enriched in homeobox genes (Figure 1). Conversely, enrichment analysis using the InterPro domain enrichment tool also showed that the set of genes specific to the brain signature (as compared to the SC signature) was enriched in terms that are not related with homeobox genes (Figure 1). 

### 2.2. In the Normal Adult Human Central Nervous System, a Set of Homeobox Genes Are Uniquely Overexpressed in the Spinal Cord as Compared to Other CNS Regions

Since the ARCHS4 library was built from the analysis of human tissues, irrespective of their pathological or normal state, we then sought to determine whether homeobox genes are indeed physiologically overexpressed in the human SC as compared to other CNS regions. To this aim, we explored the GTEx database [22,23,24] which corresponds to the currently largest repository of RNA-seq data obtained from normal human tissues. We focused our analysis on the set of homeobox genes belonging to the ARCHS4 SC-specific signatures. Starting from this list of 29 candidate homeobox genes, we retrieved the corresponding median TPM (transcripts per kilobase million) values calculated in the GTEx database from the RNA-seq analysis of spinal cord samples (*n* = 159), cerebellum (*n* = 241), hippocampus (*n* = 197), or brain cortex (*n* = 255) samples. We then filtered the results in order to retain only homeobox genes exhibiting SC mRNA levels above the threshold of 5 TPM. This approach allowed us identifying 10 homeobox genes which, under physiological conditions, are substantially expressed in the human SC but are not or only very poorly expressed in the cerebellum, hippocampus, and brain cortex (Table 1). 

It should be noted that while the brain cortex cannot be considered as a myelin-rich area, the cerebellum is abundantly myelinated and the hippocampus comprises large areas of myelinated tracts. It is thus highly unlikely that such differences may reflect differences regarding the cell composition of the SC as compared to other CNS areas. However, to address this issue, we sought to check if the HOX genes reported as poorly expressed in the brain according to the GTEx database had also been reported to be poorly expressed in cultured oligodendrocytes, astrocytes, or neurons derived from the normal human brain. To this aim, we queried the “Brain RNA-seq” database which compiles RNA-seq data obtained from primary cultures of CNS-resident cells (oligodendrocytes, astrocytes, microglia, neurons, endothelial cells) derived from adult normal human brains [25]. We found that in all analyzed cell types, the 10 homeobox genes identified as being overexpressed in the SC according to the GTEx databank exhibited mRNA levels equal to or slightly above the 0.1 FPKM threshold of detection which was set to in the “Brain RNA-seq” databank (data supplement 1). Besides the issue of cell composition, another potential drawback resides in the fact that CNS-derived samples analyzed in the GTEx database were not submitted to a careful neuropathological examination. Moreover, some of these samples were obtained from patients who died from traumatic brain injury [26]. To confirm our findings, we thus performed a complementary investigation on the previously published BNE (Brain Net Europe) transcriptomics dataset [27] from which analyzed control human CNS samples were demonstrated to be disease-free as assessed by neuropathological examination. While all the regions of interest that we explored in the GTEx database are not available in the BNE dataset, we could nevertheless assess the differential expression of homeobox genes in SC vs. brain cortex samples (Table 2). Results show that despite the relatively low number of analyzed samples in the BNE dataset (10 in each group), the retrieved expression profiles confirm the results obtained by querying the GTEx database (Table 2). 

Finally, our findings are also supported by a survey of the literature regarding the spinal cord-specific expression of homeobox genes in mice. Thus, in transgenic mice expressing β-galactosidase under the *Hoxb8* promoter, lacZ activity was shown to be restricted to the spinal cord [28]. Accordingly, *Hoxb8*-Cre (i.e., *Hoxb8*-Cre recombinase) mice were used for brain-sparing conditional gene deletion [29]. Similarly, according to the GENSAT database of engineered mouse strains [30,31], transgenic mice expressing EGFP (enhanced green fluorescent protein) under the *Hoxa5* promoter exhibit spinal cord-restricted EGFP expression. Of note, for both *Hoxb8* and *Hoxa5*, the expression pattern of reporter genes was indicative of a widespread promoter activity in both neuronal and glial cells. Finally, *Hoxb7* was demonstrated to be overexpressed by a factor >10 in SC-derived endothelial cells (cultured or freshly extracted) as compared to brain-derived endothelial cells [32].

### 2.3. The Spinal Cord-Overexpressed HOXA5 Homeobox Protein Interacts with the Gliosis-Associated Transcription Factors SOX2 (SRY-box 2) and SMAD1.

Homeobox genes encode transcription factors (TFs) which, as such, function via interacting with a wide range of proteins including TFs, co-TFs, and chromatin modifiers [33,34,35]. Indeed, complex networks of transcription factors were shown to regulate gene expression patterns in a tissue-specific manner [36]. We thus sought to identify the set of TFs establishing first shell interactions with SC-overexpressed homeobox TFs. To this aim, we queried the human proteome database “BioGrid” [37] to retrieve the most recently updated list of protein partners of spinal cord-overexpressed homeobox proteins. We obtained a list of 85 interactors (data supplement 2, sheet “HOX partners”) that we then crossed with the list of currently known human TFs [38] (data supplement 2, sheet “List Human TFs”). By this means, we retrieved 14 TFs that interact with SC-overexpressed homeobox proteins (Table 3).

Interestingly, two of these TF partners, SOX2 and SMAD1, belong to the astrocytosis-related co-expression module that we previously demonstrated in MS spinal cords (data supplement 2, sheets “MS SC gliosis-associated module” and “MS SC gliosis-associated TFs”) [9]. Both SOX2 and SMAD1 were previously shown to physically interact with HOXA5 but none of the other SC-overexpressed homeobox proteins. Considering that only 12 TFs (out of 1211 human TFs) belong to the gliosis-associated gene module identified in MS SCs, the presence of SMAD1 and SOX2 in the short list of currently known HOXA5 TF partners corresponds to an enrichment factor of 31.74 with an associated p-value of 0.002 (Fisher’s exact test). Of note, several works demonstrated the progliotic effects of SOX2 and SMAD1 [39,40,41]. Furthermore, SMAD1 belongs to the BMP (bone morphogenetic protein) and to the TGFB1 signaling pathways [42,43,44,45], both of which have been involved in astrocytosis [46,47]. Moreover, according to the BioGrid database, SOX2 and SMAD1 themselves interact with 3 TFs encoded by genes of the gliosis-associated co-expression module we identified in MS SCs: the androgen receptor (AR), GLIS3 (GLIS family zinc finger 3), and NFIB (nuclear factor I B). These findings indicate that the protein–protein interactions linking HOXA5 to SOX2 and SMAD1 are likely to be functionally relevant in the context of MS-associated spinal cord gliosis. Finally, we retrieved from the JASPAR library of predicted TF targets, the computationally inferred list of genes which harbor HOXA5 binding motifs in their promoter regions. We found that such a list of predicted targets includes *SMAD1*, *SMAD3*, *TGFB1*, and *TGFBR2*. 

### 2.4. Data Mining Analysis Indicates that the Progliotic Pathway Associated with SC-Overexpressed HOX Proteins Is Negatively Regulated by the Androgen Receptor

That AR belongs to the gliosis-associated gene module identified in MS SCs was previously proposed to reflect a failed negative feedback process. Indeed, AR exerts antigliotic effects [48,49,50,51] and was shown to potentially antagonize the TGFB1 pathway via the hijacking of SMAD3 [52] and the transcriptional silencing of *TGFB1* [53,54]. However, a global view on the potential antagonism between the AR and TGF-beta/SMAD pathway is still lacking. We thus sought to determine whether an unsupervised analysis of gene ontology (GO) terms would support the existence of such an antagonism. To achieve this goal, we queried the “QuickGO” server run by the European Molecular Biology Laboratory’s European Bioinformatics Institute (EMBL-EBI) [55,56], which allows exploring the 44,990 GO terms annotating the proteins referenced in UniProt Knowledgebase [57] across species. In particular, when querying any given GO term on the “QuickGO” server, the “Co-occurring terms” tablet provides the whole list of GO terms exhibiting similarities (i.e., shared annotated proteins) with the queried GO term. Here, we successively queried the GO terms “Positive regulation of SMAD protein signal transduction” (GO:0060391), “Negative regulation of SMAD protein signal transduction” (GO:0060392), “Negative regulation of androgen receptor signaling pathway” (GO:0060766), and “Positive regulation of androgen receptor activity” (GO:2000825). Similarities between SMAD-related GO terms and androgen receptor-related GO terms were then searched. Strikingly, only functionally antagonistic overlaps were observed, notably between the “Positive regulation of SMAD protein signal transduction” GO term and the “Negative regulation of androgen receptor signaling pathway” GO term (Table 4). 

**Figure 2:**
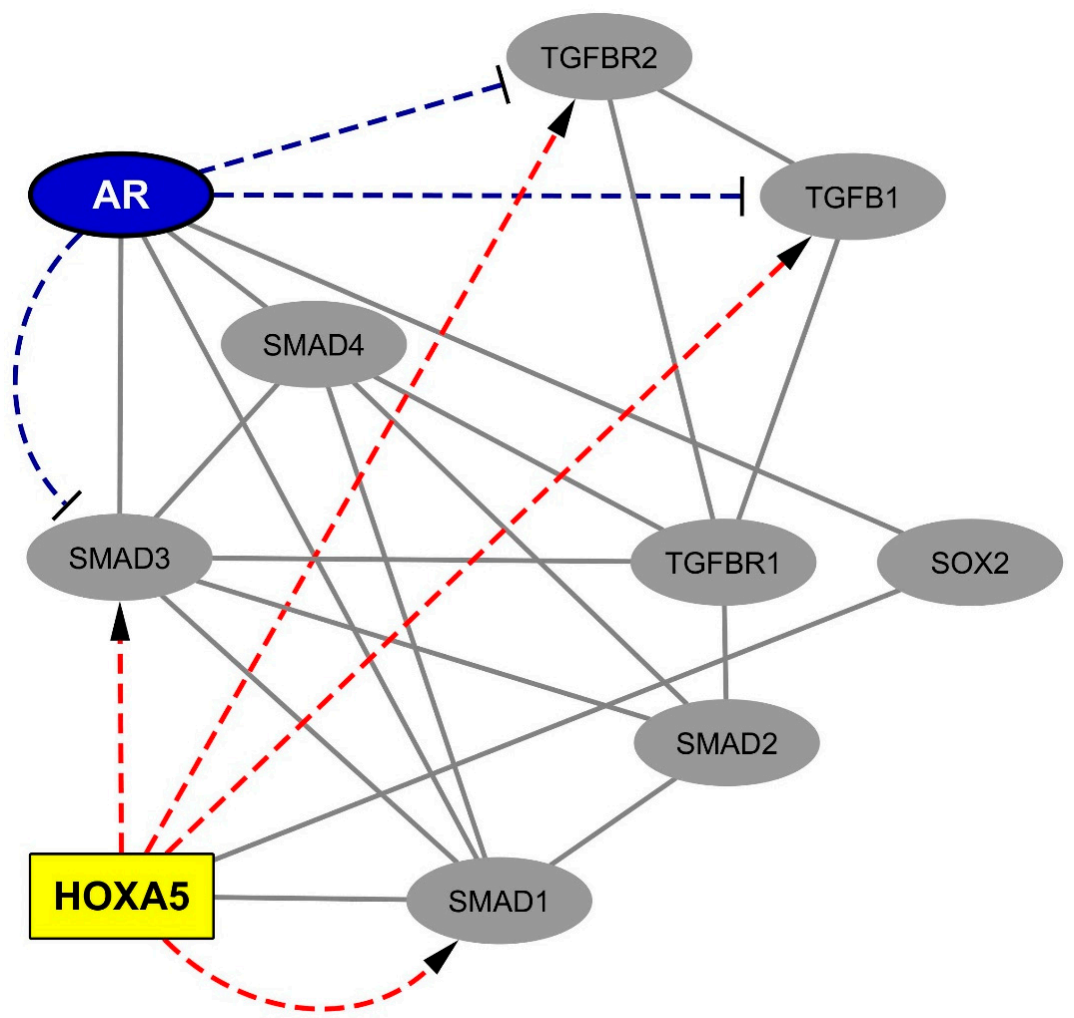
Network of physical and functional interactions linking spinal cord-overexpressed HOX proteins, members of the TGFB1 pathway, and the androgen receptor. A survey of the human proteome was performed via the BioGrid database [41] to retrieve protein–protein interactions (in grey lines) between HOXA5 (homeobox A5), SOX2 (SRY-box 2) and members of the TGFB1 pathway, i.e., SMAD1 (SMAD family member 1), SMAD2 (SMAD family member 2), SMAD3 (SMAD family member 3), SMAD4 (SMAD family member 4), TGFBR1 (transforming growth factor beta receptor 1), TGFBR2 (transforming growth factor beta receptor 2), and TGFB1 (transforming growth factor beta 1). In parallel, a survey of previously published promoter analysis data allowed retrieving and visualizing (in blue dashed lines) the robustly documented silencing effects exerted by the androgen receptor on the transcription of *SOX2*, *SMAD3*, *TGFBR2*, and *TGFB1*. Finally, the JASPAR database [58] accessed via the Harmonizome website [59] was explored in order to retrieve predicted transcriptional targets of HOXA5 (red dashed lines).

These results, obtained via an unsupervised system biology approach, further point to a functional antagonism between the SMAD signaling pathway and the AR pathway. On this basis, we then performed a manual curation of the literature and listed the promoter analysis studies which previously documented the silencing effects of AR on the transcription of *SOX2* and/or gene members of the TGF-beta pathway (data supplement 3). We were then able to build a comprehensive network gathering previously demonstrated protein–protein interactions and/or transcriptional regulatory links between AR, HOXA5, and members of the TGFB1/SMAD pathway (Figure 2). 

## 3. Discussion

### 3.1. The Spinal Cord-Overexpressed HOXA5 Gene May Amplify the TGFB1 Progliotic Pathway 

We previously proposed that, while efficiently dampening neuroinflammation, chronic overexpression of TGFB1 may promote periplaque gliosis and trigger alterations of myelin synthesis in MS spinal cords [9]. However, we did not attempt to determine whether aging and/or spinal cord-specific cues could shape such a process. We found here that under normal conditions, a unique set of homeobox genes are overexpressed in the human SC as compared to the brain. Among these, HOXA5 forms heterodimers with 2 TFs which were previously found to be involved in MS SC gliosis, namely SOX2 and SMAD1. Although, at this stage, one may only speculate on the functional impact of HOXA5 on TGFB1-mediated astrocytosis, at least two published sets of experimental data indicate that HOXA5 promotes the TGFB1 pathway: (i) in murine adipocytes, Hoxa5/Smad1 interaction induces the phosphorylation of Smad1 [60] and (ii) in human carcinoma cells, the transactivating activity of HOXA5 is crucially involved in the process of TGFB1-mediated epithelial–mesenchymal transition [61]. Finally, a re-analysis of a recent work performed in mice with experimental autoimmune encephalomyelitis (EAE) shows that widespread CNS inflammation induces a region-specific increased expression of *Hoxa5* in SC astrocytes [62]. The region-specificity of astrocyte function is a research focus of major interest that is, however, relatively poorly documented to date, especially with regard to spinal cord specificities [63,64,65,66]. Our results urge investigation of the impact of SC-overexpressed HOX proteins, notably HOXA5, on the functions of SC astrocytes under inflammatory conditions. 

### 3.2. Androgens, via Transcriptional Silencing and SMAD-Interfering Mechanisms, May Antagonize the Progliotic Effects of TGFB1 

Testosterone, the most potent androgen receptor ligand, was previously shown to promote myelin repair [67,68], exert immunosuppressive effects [69,70,71,72], and to prevent gliosis [48,49,50,51]. In male patients, accumulating data indicate a role for testosterone deficit in MS progression [73,74]. Moreover, the physiological levels of testosterone achieved in men are proposed to explain the 3:1 sex ratio observed to the disadvantage of females in RRMS incidence [73,74]. In male patients beyond age 40, such a protective effect may be progressively lost due to the premises of andropause. However, a loss of AR-mediated protection is also likely to occur in female patients since a gender-independent aging-associated decline in circulating androgen precursors is observed beyond age 40. Such androgen precursors, detectable in the blood of both males and females, are essentially synthesized by the adrenal gland and comprise dehydroepiandrosterone (DHEA), dehydroepiandrosterone sulfate (DHAS), and androstenedione [75,76,77], all of which can be metabolized in AR ligands by CNS cells including astrocytes [78]. The 1:1 sex ratio in PPMS may be thus explained by intra-CNS androgens levels which, beyond age 40, are equally insufficient in males and females with regard to the AR-mediated anti-inflammatory, anti-gliotic, and myelin repair-promoting functions. Similarly, that spinal cord-located RIS (radiologically isolated syndrome) in a male patient is associated with a higher risk of developing PPMS, and may indeed reflect a premature failure of the neuroprotective AR pathway, as we recently proposed.

### 3.3. An Imbalance between the Androgen Receptor Pathway and the HOXA5-Promoted TGFB1 Pathway May Explain the Impact of Age and Gender on MS Spinal Cord Gliosis

**Figure 3:**
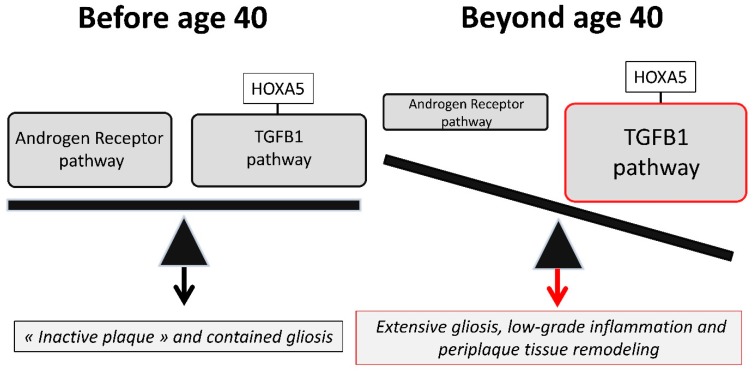
A proposed pathophysiological scheme of the fate of spinal cord lesions. In adult patients before age 40, MS spinal cord lesions resolve and repair more efficiently than brain lesions. This is due to a more potent TGFB1 anti-inflammatory pathway which is favored by physiological spinal cord overexpression of the SMAD1-interacting protein HOXA5. At this stage, the androgen receptor (AR) anti-inflammatory pathway superimposes to the TGFB1 pathway, promotes myelin repair, and balances the progliotic effects exerted by TGFB1. However, in adult patients beyond age 40, the physiological decline in AR ligands provokes an imbalance between the AR and the TGFB1 pathways. Neuroinflammation mediated by blood-derived immune cells is maintained at relatively low levels but AR-mediated myelin repair becomes inefficient. The imbalanced TGFB1 pathway fuels extensive gliosis and leads to profound alterations of myelin homeostasis which eventually translate into a process of myelinodegeneration [9].

As revealed earlier, the SC-overexpressed HOXA5 protein may favor the TGFB1-mediated anti-inflammatory pathway in MS spinal cords, which explains the favorable outcome of SC MS plaques regarding their inflammatory activity. Moreover, until the aging-related decline in AR ligands, a proper balance between the AR-mediated antigliotic pathway and the TGFB1-mediated progliotic pathway may limit gliosis in MS SCs. However, we propose that due especially to the SC overexpression of HOXA5, the physiological decline of AR ligands beyond age 40 exposes MS spinal cords to the long term deleterious effects of TGFB1. Indeed, as supported by the molecular histology of SC periplaques [8,9], TGFB1 may fuel a process of extensive astrocytosis that possibly stems from pre-existing sites of plaque-associated gliosis (Figure 3). 

Such a progliotic TGFB1 anti-inflammatory pathway may be triggered by persisting low-grade inflammation or, alternatively, may self-perpetuate. We previously provided data from gene co-expression analyses indicating that TGFB1, along with a limited set of cytokine, may alter myelin homeostasis in MS spinal cords [9]. In support of this observation, TGF-beta was recently demonstrated to not only render aging microglia inhibitory to myelin repair but to significantly block the differentiation of oligodendrocyte precursor cells into mature myelinating oligodendrocytes [17]. Finally, it is worth noting that increased levels of TGFB1 were previously reported in the cerebrospinal fluid of MS patients as compared to controls [79]. The data mining results exposed in the present paper point to a molecular crosstalk between the AR and the TGFB1 pathways. At the protein level, previously published biochemical analyses showed that AR interaction with the TGFB1 signaling molecule SMAD3 was responsible for an antagonism between the AR and the TGFB1 pathways [52,80,81,82]. Also at the transcriptional level, several papers demonstrated the AR-mediated silencing of key genes of the TGFB1 pathway (data supplement 3), including SMAD3 [83]. Since SMAD3 is involved in the progliotic effects of TGFB1 [84,85], one may hypothesize that the hijacking of SMAD3 by AR may support the antigliotic effects exerted by androgens in different in vivo experimental settings [48,49,50,51,67,86]. Finally, since TGFB1 and AR signaling pathways may antagonize each other, any imbalance to the detriment of the AR pathway may also result in a lowered ability of AR ligands in promoting myelin repair and dampening inflammation.

### 3.4. Therapeutic Implications

There are two categories of arguments indicating that clinically relevant therapeutic implications may be drawn from the notion of TGFB1/AR imbalance in MS progressive forms.

Point 1: Blocking the TGFB1 pathway or stimulating the AR pathway efficiently delays and/or dampens EAE. Regarding TGFB1, in-depth mechanistic investigations were performed in at least 2 major articles showing that blocking the TGFB1 pathway is therapeutically effective in the context of EAE [87,88]. In particular, Luo et al. [88] demonstrated (i) an early and sustained upregulation of TGFB1 expression by microglia and astrocytes in EAE mice, (ii) a parallel intra-CNS induction of the TGF-beta signaling pathway and (iii) substantial clinical and histological benefits afforded by the systemic administration of a TGF-beta receptor 1 (TGFBR1) inhibitor. In another key paper, Lanz et al. [87] showed that in EAE mice (i) endogenously generated angiotensin II sustains inflammation via the glial upregulation of TGFB1 and (ii) the systemic administration of a pharmacological inhibitor of angiotensin II type 1 receptor dampens clinical signs and blunts intra-CNS T-cell infiltration via a downregulation of the TGFB1 pathway. Conversely, in transgenic mice overexpressing TGF-beta 1 under the control of an astrocyte-specific promoter, EAE develops earlier and is more severe than in wild-type littermates [89]. With regard to the AR pathway, the systemic administration of testosterone was shown to exert anti-inflammatory and neuroprotective effects in male EAE mice [90,91] and in female EAE mice [92]. Interestingly, in a rat model of chronic EAE, therapeutic effects of testosterone were accompanied by a marked decrease of spinal cord astrocytosis [51]. Of note, a similar protective activity in the EAE model was reported with the androgen precursors DHEA [93] and androstenedione [94]. 

Point 2: Molecules that either block the TGFB1 pathway or stimulate the AR pathway have market authorization and are well tolerated. TGFB1 is recognized to play a major pathophysiological role in systemic sclerosis, a chronic inflammatory and autoimmune disorder affecting the connective tissue [95]. In patients suffering from systemic sclerosis, the anti-TGFB monoclonal antibody fresolimumab, which targets the 3 TGFB isoforms (TGFB1, TGFB2, and TGFB3), was shown to be well tolerated and clinically efficient in a phase II clinical trial [96]. Fresolimumab is considered as a promising therapy of systemic sclerosis, either as a standalone treatment or in combination with classical immune-targeting compounds such as rituximab or mycophenolate mofetil [97]. Interestingly, fresolimumab is likely to cross a partially compromised blood–brain barrier as shown in patients suffering from glioblastoma [98]. Also of note, a large array of pharmacological inhibitors of the TGF-beta pathway have been developed and are being tested in phase I or phase II trials for the treatment of solid tumors [99]. Finally, inhibitors of the renin–angiotensin system are off-the-shelf compounds that were previously shown to efficiently antagonize the TGFB1 pathway [100,101,102,103,104]. One of these compounds, the angiotensin II receptor type 1 antagonist Losartan, was shown to efficiently prevent the pathogenic intra-CNS overproduction of TGF-beta 1 involved in post-stroke epilepsy [105] and in Alzheimer’s disease [106]. However, on the basis of our results, inhibiting the TGF-beta 1 pathway may become effective only if the AR pathway is concomitantly restored. In this regard, phase I/II trials in RRMS male patients showed promising neuroprotective and immunomodulatory effects of testosterone as well as a good tolerance [107,108,109]. On the other hand, testosterone treatment is likely to expose patients to potentially harmful adverse effects in the long term. An alternative possibility would be the use of the androgen precursor DHEA which, using a large cohorts of subjects, has been proven to be safe in long-term treatments, irrespective of gender [110,111,112]. Again, one should keep in mind that the antigliotic activity of AR ligands may be antagonized by TGFB1. Hence, if not associated with a therapeutic strategy aimed at dampening the impact of chronic TGFB1 overproduction, the use of AR ligands may fail to block gliosis and low-grade inflammation. Supporting this view, EAE in middle-age male mice is characterized by a chronic rather than acute clinical course and an overall unresponsiveness to testosterone therapy [113].

## 4. Materials and Method

All bioinformatics and data mining analyses were performed at least 3 times between January 2018 and November 2019.

### 4.1. Workflow of the Study

**Figure 4:**
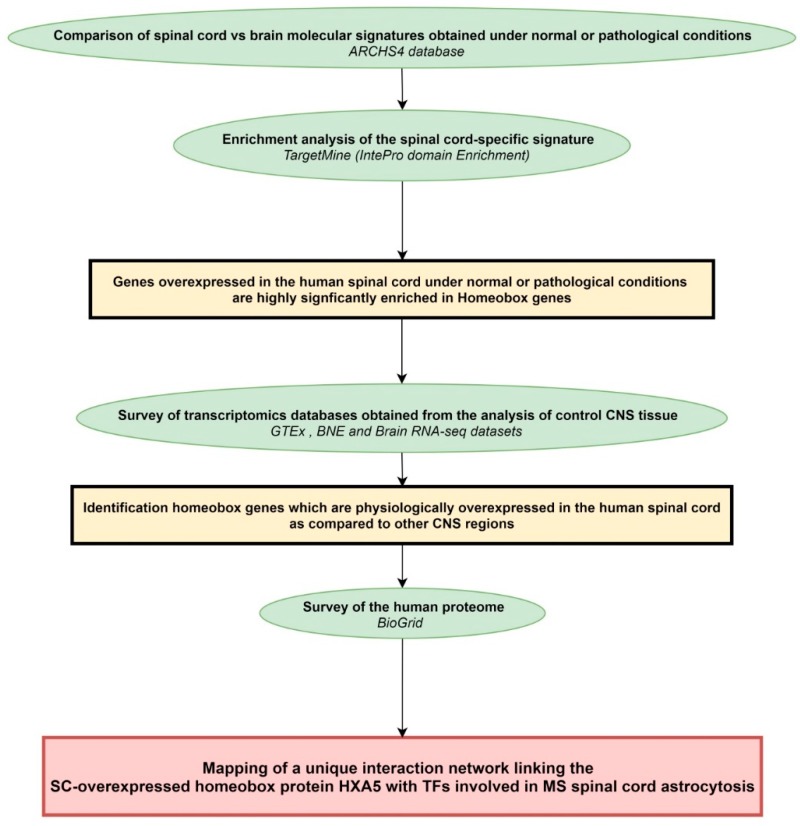
Workflow of the study. Rectangles in yellow or red frame the main results obtained following each of the analytical steps briefly described in green ellipse shapes. Terms in italics correspond to the name of the bioinformatics tools used for each analytical step. BNE: Brain Net Europe; GTEx: genome-tissue expression; SC: spinal cord; TFs: transcription factors.

The general workflow of the present study is shown in Figure 4.

### 4.2. Mining of Transcriptomics Databases Obtained from the Analysis of Human CNS Tissue Samples

The ARCHS4 database gathers and combines 84,863 sets of publicly available human RNA-seq data for which gene expression values were z-score normalized across samples [19]. From these data, genomic signatures were obtained for 108 human tissues or cell types, irrespective of the presence or absence of a pathological state. The ARCHS4 library of genomic signatures was retrieved from the Enrichr platform [18] and SC vs. brain signatures were crossed in order to identify a list of genes which, as compared to the brain, are specific to the SC signature. In parallel, we explored the GTEx mRNA expression database [22,23,24], which corresponds to the currently largest repository of RNA-seq data obtained from normal human tissues. From the GTEx databank, we retrieved the median TPM (transcripts per kilobase million) values obtained from the analysis of spinal cord samples (*n* = 159), cerebellum samples (*n* = 241), hippocampus (*n* = 197), or brain cortex (*n* = 255). Finally, as a confirmatory investigation, we also performed a survey of the Brain Net Europe (BNE) dataset [27], gathering transcriptomics data obtained from the analysis of pathological vs. control human CNS samples which had been carefully selected on the basis of neuropathological examination [27]. In this study, the authors concurrently performed expression profiling of 118 samples from 6 distinct CNS regions derived from control patients affected by non-CNS disorders or by patients affected by one of the following CNS diseases: multiple sclerosis, schizophrenia, Huntington’s disease, Parkinson’s disease, Alzheimer’s disease or amyotrophic lateral sclerosis. From this study, of which the results are deposited in the publicly available databank “GEO DataSets” (GSE26927), we retrieved the RNA expression data obtained from 10 normal human SC samples (including 3 replicates) and 10 normal brain cortical (BC) samples (subpial grey matter from the frontal gyri). We then performed differential expression analysis of SC vs. BC mRNA profiles using the unequal variance and bilateral Student’s *t* test (i.e., a Welch test) adjusted for multiple test corrections using the Bonferroni procedure. Concurrently the same analysis was conducted using “GEO2R”, a NCBI interactive online tool specifically designed to compare RNA expression data archived in GEO datasets [24]. 

### 4.3. Mining of the Proteomics Database BioGrid

BioGrid is a public database that archives protein interaction data obtained by high- or low-throughput experimental approaches. Interactors of specific HOX proteins were individually retrieved on BioGrid. Filters applied in our search allowed excluding protein–protein interactions that had been reported in non-human species as well interactions that were not published. Results were filtered for transcription factors (TFs) by crossing the retrieved list of interactors with the list of currently known human TFs [38].

### 4.4. Enrichment Analyses

Enrichment analysis tools and corresponding tasks performed in this study are described below. 

The enrichment web platform TargetMine [20]: TargetMine is a monthly updated web platform that allows performing enrichment analysis of 9 large libraries of gene/protein lists. We used the InterPro domain enrichment tool which exploits the InterPro protein families database [21]. The TargetMine bioinformatics platform provides *p*-values computed from the Fisher’s exact test and adjusted for multiple test corrections using notably the Benjamini and Hochberg procedure. 

The gene ontology and GO annotations “QuickGO” website of the European Molecular Biology Laboratory’s European Bioinformatics Institute (EMBL-EBI) [55,56]. The “QuickGO” web server, run by the European Molecular Biology Laboratory’s European Bioinformatics Institute (EMBL-EBI) [55,56], allows exploration of the 44,990 GO terms used to annotate the proteins referenced in the UniProt Knowledgebase [57] across species. When querying any given GO term on the “QuickGO” server, the “Co-occurring terms” tablet provides the whole list of GO terms exhibiting similarities (i.e., shared annotated proteins) with the queried GO term. We used this webtool to search for similarities between SMAD-related GO terms and androgen receptor-related GO terms.

The Harmonizome website [59]: Harmonizome is a regularly updated website which compiles a large collection of processed biological datasets obtained by multiple experimental and/or computational approaches. From any queried biological object (gene symbol, protein name, biological process, etc.) sets of associated biological objects may be retrieved, allowing for the generation of integrated knowledge on the queried object. Here, we used the JASPAR library [58] to retrieve the predicted targets of HOXA5.

## 5. Conclusions

The present study, based on the mining of transcriptomics and proteomics data, addresses a yet poorly explored connection between age, gender, and spinal cord in MS progressive forms [114]. We conclude that the aging-related decline in AR ligands might be responsible for a process of TGFB1-mediated gliosis that preferentially targets MS spinal cords. Such a spinal cord bias is, at least in part, determined by the constitutive overexpression of homeobox genes (notably HOXA5) in the human spinal cord as compared to the human brain. We propose that MS patients suffering from a progressive form of the disease might benefit from a treatment strategy aimed at both dampening the TGFB1 progliotic pathway and promoting the AR antigliotic pathway.

## Figures and Tables

**Table 1:** Region-specific homeobox mRNA median TPM (transcripts per kilobase million) values retrieved from the GTEx databank [20,21,22].

	Spinal Cord (*n* =159)	Cerebellum (*n* = 241)	Hippocampus (*n* = 197)	Brain Cortex (*n* = 255)
HOXA2	8.14	0	0	0
HOXA4	29.14	0.03	0	0.01
HOXA5	23.01	0.08	0.01	0.02
HOXB2	20.31	0.54	0.19	0.10
HOXB3	6.16	0.17	0.05	0.04
HOXB5	8.52	0	0	0
HOXB6	5.37	0	0	0
HOXB7	15.82	0	0.05	0.02
HOXB8	17.91	0	0	0
HOXD8	5.41	0	0	0.01

**Table 2:** Comparisons of spinal cord vs. brain cortex mRNA levels of homeobox genes retrieved from the BNE dataset [23].

Gene Symbol	SC/BC Ratio	Adjusted *p*-Value
*HOXA2*	15.49	4.57 × 10^−7^
*HOXA4*	58.62	2.62 × 10^−6^
*HOXA5*	234.90	9.03 × 10^−6^
*HOXB2*	41.46	1.14 × 10^−7^
*HOXB3*	2.58	0.01
*HOXB5*	52.41	0.002
*HOXB6*	1.74	0.06
*HOXB7*	102.29	2.47 × 10^−6^
*HOXB8*	407.52	0.009
*HOXD8*	13.42	0.001

**Table 3:** List of transcription factors which physically interact with spinal cord-overexpressed HOX proteins.

SC-Overexpressed HOX Genes	Gene Symbols (Bold) and Names of TFs Interacting with SC-Overexpressed HOX Proteins
*HOXA5*	***DDIT3*** (DNA damage inducible transcript 3)
*HOXA5*	***FOXA2*** (forkhead box A2)
*HOXA5*	***FOXO1*** (forkhead box O1)
*HOXA5*	***SMAD1*** (SMAD family member 1)
*HOXA5*	***SOX2*** (SRY-box 2)
*HOXA5*	***TWIST1*** (twist family bHLH transcription factor 21)
*HOXA5*	***ZNF707*** (zinc finger protein 707)
*HOXB5*	***HOXC4*** (homeobox C4)
*HOXB5*	***HRAS*** (HRas proto-oncogene, GTPase)
*HOXB5*	***NKX2-1*** (NK2 homeobox 1)
*HOXB6*	***ALX4*** (ALX Homeobox 4)
*HOXB6*	***TCF21*** (transcription Factor 21)
*HOXB7*, *HOXB8*	***PBX1*** (PBX homeobox 1)
*HOXB8*	***PBX3*** (PBX homeobox 3)

**Table 4:** Antagonistic overlap between SMAD-related GO terms and androgen receptor-related GO terms.

Queried GO Term	Relevant Overlapping GO Term	Rank of Similarity
Positive regulation of SMAD protein signal transduction (GO:0060391)	Negative regulation of androgen receptor signaling pathway (GO:0060766)	23rd from 743 overlapping GO terms
Negative regulation of SMAD protein signal transduction (GO:0060392)	No overlap with androgen receptor-related GO-term	Not applicable
Negative regulation of androgen receptor signaling pathway (GO:0060766)	Positive regulation of SMAD protein signal transduction (GO:0060391)	4th from 562 overlapping GO terms
Positive regulation of androgen receptor activity (GO:2000825)	No overlap with SMAD-related GO-term	Not applicable

## Supplementary Materials

Supplementary materials can be found at https://www.mdpi.com/1422-0067/20/23/5934/s1. Data Supplement 1: RNA-seq data were retrieved from the “brain RNA-seq” web portal (http://www.brainrnaseq.org/). Data are expressed in Fragments Per Kilobase Million, Data Supplement 2: List of human protein partners of SC-overexpressed Homeobox proteins, Data Supplement 3: List of publications demonstrating the AR-mediated transcriptional repression of human genes previously demonstrated to promote astrocytosis.
