# Supplementary material for: TGFB1-Mediated Gliosis in Multiple Sclerosis Spinal Cords Is Favored by the Regionalized Expression of HOXA5 and the Age-Dependent Decline in Androgen Receptor Ligands
Source: Int J Mol Sci. 2019 Nov 26;20(23):5934. doi: 10.3390/ijms20235934 (PMC6928867; doi:10.3390/ijms20235934)
Supplement: Supplementary file 1 [file ijms-20-05934-s001.zip › Data supplement 3.docx]

**List of publications demonstrating the AR-mediated transcriptional repression of human genes previously demonstrated to promote astrocytosis**

| **AR-repressed target gene** | | **Publication** |
| --- | --- | --- |
| *TGFB1* | * Negative androgen-response elements mediate androgen-dependent transcriptional inhibition of TGF-β1 and CDK2 promoters in the prostate gland. *J Androl* 2012 Jan-Feb;33(1):27-36.* Transcriptional regulation of the TGF-beta1 promoter by androgen receptor. *Biochem J* 2008 Dec 15;416(3):453-62. |  |
| *TGFBR2* | * Androgenic control of transforming growth factor-beta signaling in prostate epithelial cells through transcriptional suppression of transforming growth factor-beta receptor.  *Cancer Res* 2008 Oct 1;68(19):8173-82 |  |
| *SMAD3* | * DHT selectively reverses Smad3-mediated/TGF-beta-induced responses through transcriptional down-regulation of Smad3 in prostate epithelial cells. *Mol Endocrinol* 2010 Oct;24(10):2019-29 |  |
| *SOX2* | * Sox2 is an androgen receptor-repressed gene that promotes castration-resistant prostate cancer. *PLoS One* 2013;8(1):e53701. |  |
